# Virtual visits: Friend or foe of patient-centred care?

**DOI:** 10.23889/ijpds.v1i1.392

**Published:** 2017-04-19

**Authors:** Kim Mcgrail

**Affiliations:** 1 University of British Columbia

## Background

Patient-initiated virtual visit consultations in primary care are a new form of care that is increasingly available in parts of Canada. There is a need to develop an understanding of patient perspectives on virtual visits and how they affect patient experience, access to care, and health services utilization patterns from a system perspective. This paper will shed some light on this new form of patient care delivery.

## Approach

We accessed fee-for-service physician payment data, patient and physician demographic data, and PharmaNet prescription data, from 2010/11-2013/14. We examined overall utilization of GP virtual visits defined by BC physician billing codes to understand the characteristics of providers and patients providing and using these services. We assessed the relationship of virtual visits with other types of care including referrals to specialist services, medication prescribing, and overall costs of physician care. We used a matched cohort and time series analysis to answer the question of whether virtual visits displace or add to other forms of patient care.

## Main Findings

While the growth in virtual visits has been rapid in BC, these services still represent a very small proportion of overall primary care. There are patient users in urban and rural regions of BC, but less than 1% of the BC population has had a virtual visit. Users of virtual visits tend to be younger and use is more likely for people with one or more major conditions (measured by ADGs). Only 144 primary care GPs (out of close to 5,000 in BC) provided virtual visits in 2013/14. About one-third of patients have a virtual visit with a physician already known to them, with the rest seeing a physician for the first time during their virtual visit. These visits do not appear to add costs to care, though there is a suggestion that it they are more effective as part of an ongoing therapeutic relationship. 

## Conclusion

Virtual visits are a small portion of total primary care but are expected to increase. In some cases virtual visits appear to supplement existing forms of access, offering a new means by which to interact with a known provider. In other cases virtual care is with a new provider, which may suggest walk-in clinic type of service use. The implications of these two scenarios are clearly different, and further research with longer follow-up will be helpful in understanding long-term implications.

